# Brief Cognitive Analytic Therapy (CAT)‐Informed Reformulation for Young People With Eating Disorders: A Case Series

**DOI:** 10.1002/cpp.70043

**Published:** 2025-04-02

**Authors:** C. Green, G. Mannion, I. Gill, S. Hartley, B. J. Dunlop, P. J. Taylor

**Affiliations:** ^1^ Division of Psychology and Mental Health, School of Health Sciences, Faculty of Biology, Medicine and Health University of Manchester Manchester UK; ^2^ Greater Manchester Mental Health NHS Foundation Trust Manchester UK; ^3^ Pennine Care NHS Foundation Trust Ashton‐Under‐Lyne UK; ^4^ Lancashire and South Cumbria NHS Foundation Trust Preston UK

**Keywords:** adolescents, brief reformulation, cognitive analytic therapy, eating disorders, young adults

## Abstract

**Background:**

Onset of Eating Disorder (ED) peaks in young people, and interpersonal factors can influence development and maintenance. With increased referrals to ED services, accessible, brief interventions may support early intervention and improve outcomes. Cognitive Analytic Therapy (CAT) is a transdiagnostic relational approach, which can offer benefit for a range of presenting difficulties. This study aimed to assess the feasibility and acceptability of a brief, CAT‐informed reformulation for young people with ED.

**Design:**

A case series design recruited eight young people who met inclusion and exclusion criteria to participate in the five‐session reformulation intervention.

**Method:**

Recruitment took place from NHS ED services. Feasibility and acceptability were measured via recruitment, retention, qualitative feedback and missing data. Psychological distress, ED severity, personal recovery and motivation to change were assessed at baseline, post‐intervention and follow‐up. Participants also completed sessional measures of psychological distress and alliance.

**Results:**

Eight eligible participants aged 15–24 years (M = 20.25, *SD* = 3.58) consented to take part and received the intervention. All participants attended all intervention sessions and completed all assessments. Participants reported positive experiences of the intervention. There was an associated reduction across clinical outcomes, including psychological distress and ED severity.

**Conclusions:**

This case series showed promising results on the feasibility and acceptability of a brief CAT‐informed reformulation for young people with ED. However, the study had a small sample size and no comparator control group. Larger scale exploration of a brief CAT‐informed reformulation for EDs among young people is warranted.

**Clinical Trial Registration:**

The study was preregistered with clinicaltrials.gov (NCT05746364).

**Summary**

- This case series supports the acceptability and feasibility of a brief CAT‐informed reformulation for young people with ED, suggesting further exploration on a larger scale is warranted.
- The reformulation intervention had good referral rates, excellent retention to treatment and outcome measure completion. Young people shared positive feedback of the reformulation and generally found it acceptable.
- The largest improvements were found in psychological distress and ED severity. Small improvements were also observed in readiness to change and recovery.

## 1 Introduction

Eating disorders (EDs) are a global concern with estimated lifetime mean prevalence rates of 8.4% for cisgender women and 2.2% for cisgender men (Galmiche et al. 2019). Characterised by severe and enduring psychological distress associated with disturbances in eating and weight‐related behaviours, EDs can involve restricted eating, binge eating, vomiting and excessive exercise (American Psychiatric Association; APA 2023; National Institute for Health and Care Excellence; NICE 2017). EDs are associated with significant wider social, mental, cognitive and physical health implications (Campbell and Peebles 2014; Lindstedt et al. 2018; Rosen 2003; Thomas, Vartanian, and Brownell 2009), alongside significant medical risks, including death (Arcelus et al. 2011). While most anorexia nervosa (AN) deaths are directly associated with disorder‐related medical complications (Zipfel et al. 2015), higher rates of suicide and self‐harm are also present across EDs (Kostro, Lerman, and Attia 2014). Risk of onset peaks in adolescents and young adults (15–25; NICE 2017; Schmidt et al. 2016), and indicators suggest onset is starting increasingly younger in Western countries (Favaro et al. 2009; Swanson et al. 2011). As early intervention can improve clinical outcomes and weight recovery (Allen et al. 2023; Fukutomi et al. 2020), effective treatments of EDs in young people are a priority.

Currently, family‐based and behavioural approaches have been the main focus for treating young people with EDs. Family‐based treatment (FBT) is the frontline evidence‐based treatment (EBT) for children and adolescents with AN and bulimia nervosa (BN; NICE 2017). Despite research suggesting FBT is effective for many, approximately 60% of adolescents with AN or BN do not reach full remission (Lock and Le Grange 2019). In such instances, suggested interventions include cognitive behaviour therapy (CBT) or adolescent‐focused psychotherapy (AFP; NICE 2017). Randomised control trials (RCTs) show such approaches can be efficacious (Atwood and Friedman 2020; Lock et al. 2010). However, overall estimates of recovery for adolescents with EDs remain at approximately 30%–40% (Datta et al. 2023; Lock 2015). Regardless of benefit, for some young people, FBT approaches are not possible, for example, if parental or carer involvement is limited. Furthermore, others may not be ready to engage in a change‐focused intervention such as CBT. Research has an important role in investigating alternative approaches that might benefit young people with EDs. Given the current focus on behavioural and family‐based approaches, it is important that alternative treatments are also investigated. Ultimately, this will help to expand the available repertoire of evidence‐based interventions for young people with EDs, allowing for greater service flexibility and patient choice.

Research shows that ED risk is influenced by a combination of genetic factors (Trace et al. 2013) and wider sociocultural or environmental conditions (Bakalar et al. 2015). As such, interpersonal processes alone are unlikely to be sufficient to contribute to ED onset, but they may play a role in the development and/or maintenance of the conditions. For example, the presence of an overcontrolling, indifferent or abusive caregiver or bullying may impact ED development (Connan et al. 2007; Copeland et al. 2015). ED behaviours may also serve intrapersonal emotion regulation functions (Brockmeyer et al. 2014), although these functions can relate to negative emotions experienced within specific interpersonal contexts (Henderson et al. 2019). ED behaviours may be learnt as a means to avoid interpersonal expression of emotion, or cope with relational conflict (Arcelus et al. 2013), but may also themselves maintain or increase interpersonal difficulties, including conflict (Sim et al. 2009). As a reported precursor and consequence of ED, understanding and working within the relational contexts of EDs is vital.

One distinctly relational approach, which may offer benefit across adolescents and young adults with EDs, is cognitive analytic therapy (CAT; Ryle and Kerr 2020). CAT is a transdiagnostic model that proposes early relational experiences are internalised and inform self‐to‐self relating and how the self relates to others (internalised reciprocal roles). As a result of internalised roles, individuals develop goal‐orientated procedures to move towards desired relational positions and away from aversive positions. ED behaviours may emerge within procedures; for example, an individual experiencing relational condition in which they feel ‘not good enough’ may continuously strive for thinness to gain a desired state of acceptance. Due to the absolute nature of desired states (e.g., *complete* acceptance or *perfect* care), they are often unattainable or unsustainable; thus, procedures ultimately function to maintain distress. CAT aims to bring these processes into an individual's conscious awareness through a shared and understandable description (reformulation), recognition of patterns within and outside of sessions and revision of patterns through new procedures (‘exits’).

Previous NICE ED guidance (NICE 2004) referenced the potential value of CAT for AN. Recommendations stemmed from two RCTs conducted with adults with EDs (Treasure et al. 1995; Dare et al. 2001). Although both had small sample sizes, CAT showed promising results; those that completed CAT rated themselves as having improved significantly more than those that completed educational behavioural therapy (*N* = 14; Treasure et al. 1995), and outcomes from CAT did not differ significantly compared with other evidence‐based approaches including focal psychotherapy and family therapy (*N* = 22; Dare et al. 2001). However, updated NICE guidelines (2017) removed this consideration following a lack of further controlled trials. This highlights the need for further research into CAT and CAT‐informed approaches. There has been a general lack of larger scale RCTs into CAT, with the evidence base instead including more practice‐based evidence (Calvert and Kellett 2014). However, a meta‐analysis of 25 studies utilising CAT for a range of presenting difficulties, including anxiety and complex trauma, demonstrated moderate–large pre–post reductions in interpersonal difficulties, global functioning and depression (Hallam et al. 2021). Another meta‐analysis suggested that across a range of conditions, CAT had lower rates of dropout (weighted mean = 18.69%) than other approaches, such as cognitive behavioural therapy (weighted mean = 26.2%) and dialectical behaviour therapy (weighted mean = 28.0%; Simmonds‐Buckley et al. 2022). Furthermore, as a transdiagnostic approach, CAT may offer value in ED intervention due to high levels of comorbid needs, which may require a more adaptive treatment (Keski‐Rahkonen and Mustelin 2016; de Jonge et al. 2003).

Given the near two‐third rise in referrals to National Health Service (NHS) Children and Adolescent Mental Health Service (CAMHS) ED services since 2019 (NHS England 2022), brief psychological approaches may also meet the needs of those on waitlists. A recent feasibility study of a brief five‐session CAT‐informed reformulation‐focused approach for young people who self‐injure (cognitive analytic therapy for containing self‐harm in young people; CATCH‐Y; Haw et al. 2024) has found this acceptable. Following this case series, alongside emerging support for CAT‐informed interventions for mental health difficulties in young people more broadly (Chanen et al. 2008), CATCH‐Y was adapted for use in EDs among young people (relational reformulation for difficulties with eating; RIDE). RIDE is a brief five‐session CAT‐informed reformulation for young people who struggle with ED. The RIDE reformulation should enhance the efficacy of longer‐term interventions if still clinically indicated. CAT‐informed reformulations have shown promise with this aim; for example, a brief CAT‐informed assessment for self‐harm in adolescents improved subsequent treatment engagement over 2 years (Ougrin et al. 2013). Like this approach, RIDE has a greater focus on building understanding (reformulation) with less time spent on revision. The primary aim of RIDE is to increase motivation and confidence towards recovery. We therefore foresee RIDE operating as an initial intervention that is offered to young people when referred to a service, which can allow initial intervention whilst they await longer‐term treatment, generate motivation around therapeutic work and develop an initial understanding of a young person's ED and surrounding difficulties that could inform future work. As a CAT‐informed approach, RIDE could be delivered by a range of different practitioner groups, increasing its ease of implementation into services.

In accordance with the Medical Research Council (MRC) and National Institute for Health Research (NIHR) Complex Intervention Framework (2019, as cited in Skivington et al. 2021), this study was a case series that evaluates the feasibility and acceptability of RIDE. Feasibility was measured through recruitment rates, retention rates and data completeness. Acceptability was assessed by attendance of participants and qualitative feedback. The secondary aim was to investigate whether there was preliminary evidence for change across measures of psychological distress, eating disorder pathology, motivation to change and personal recovery.

The following hypotheses around feasibility and acceptability were tested: (i) over 50% of those referred to the study and eligible to participate would consent to take part; (ii) at least 70% of those recruited would attend all five sessions; and (iii) the level of missing data on clinical outcome measures at assessments (for those still retained in the study) would not exceed 20% per assessment.

## 2 Method

### 2.1 Design

The study was a small‐scale pilot study of a CAT‐informed reformulation (RIDE) utilising pre and postreformulation assessments. The study was preregistered with clinicaltrials.gov (NCT05746364). This research was granted approval from the University of Manchester Sponsorship panel and the NHS Research Ethics Committee (REC reference: 23/YH/008). Recruitment took place within four local NHS trusts who also issued local approval.

### 2.2 Participants

As CAT is a transdiagnostic approach and many behavioural and cognitive features of specific ED diagnoses (such as overvaluation of weight or shape) are transdiagnostic across EDs (Castellini et al. 2011; Mitchison and Mond 2015), this research focused broadly on ED. Participants were included if they were (a) between 14 and 25 years old and (b) under the care of a local NHS ED service with an allocated clinician. Participants were excluded if they (a) were currently receiving an alternative one‐to‐one psychological therapy or FBT (not including low‐intensity family support or skills‐based group); (b) had a moderate to severe intellectual disability, which would have impaired their ability to participate without considerable adaptations, as judged by the young person or the clinical team; (c) had inadequate English‐language speaking skills due to limitations in their ability to engage with talking therapies in the English language; and (d) were judged to be at high risk of harm to themselves, operationalised as having current suicidal thoughts with a high intent, an active plan to end their life or a sustained trajectory of recent weight loss.

Consistent with guidelines concerning the development of evidence for complex interventions, it is recommended that feasibility and acceptability of interventions be investigated on a smaller scale prior to larger scale evaluations (e.g., Skivington et al. 2021). Given that there has been no prior investigation of RIDE we aimed to initially pilot this approach in a small sample. This is consistent with the field where initial small‐scale investigations focused on feasibility are undertaken prior to further evaluation (e.g., *n* = 7–15; Federici and Wisniewski 2013; Haw et al. 2024; Knagg et al. 2022; Taylor et al. 2019). A sample size of nine was sought, as this would allow a preliminary assessment of feasibility and acceptability. As the focus was on feasibility and inferential tests were not planned, a power calculation was not required.

### 2.3 Measures

#### 2.3.1 Acceptability and Feasibility Outcome Measures

An adapted version of the Experience of Service Questionnaire (ESQ; Brown et al. 2014) was used to gain feedback and measure aspects of the acceptability following completion of the intervention. The ESQ was developed to measure satisfaction with CAMHS services and was adapted to measure experiences of therapy only. Seven statements regarding the therapy sessions were presented, and the young people were asked to state how true each statement was from the options: ‘certainly true’, ‘partly true’, ‘not true’ and ‘do not know’. There was also space to comment directly on their experience of the reformulation sessions.

#### 2.3.2 Clinical Outcome Measures

Outcome measures relating to disordered eating, psychological distress, therapeutic relationship and functional recovery were used to assess preliminary support for the effectiveness of the intervention. Clinical outcome measures administered timepoints can be seen in Table 1.

**TABLE 1:** Clinical outcome measures and timepoints.

Measure	Preintervention	Weekly	Postintervention	Follow‐up (2 weeks postintervention)
EDE‐Q	✓		✓	
YP‐CORE	✓	✓	✓	✓
ReQuest‐YP	✓		✓	✓
URICA	✓		✓	✓
Adapted ESQ			✓	
SRS		✓		

Abbreviations: EDE‐Q, Eating Disorder Examination Questionnaire; ESQ, Experience of Service Questionnaire; ReQuest‐YP, Recovery Questionnaire for Young People; SRS, Session Rating Scale; URICA, University of Rhode Island Change Assessment Scale; YP‐CORE, Young Person's Clinical Outcome in Routine Evaluation.

*The Eating Disorder Examination Questionnaire (EDE‐Q)*: The EDE‐Q was used to assess the range and severity of features associated with a diagnosis of eating disorder using four subscales (restraint, eating concern, shape concern and weight concern) and a global score (Fairburn and Beglin 1994). Good internal consistency has been demonstrated for the global score (α = 0.95; Aardoom et al. 2012) and across subscales (α = 0.88–0.96; White et al. 2013). A cut‐off score of ≥ 2.7 was used to indicate clinical cut off (Jebeile et al. 2024; Rø, Reas, and Stedal 2015).

*The Young Person's Clinical Outcome in Routine Evaluation (YP‐CORE; Twigg et al*. 2009 *)*: A 10‐item questionnaire measuring psychological distress in young people. An evaluation of the YP‐CORE showed good internal and retest reliability (α = 0.85, r = 0.76; Twigg et al. 2016).

*Session Rating Scale (SRS)*: A four‐item rating scale measure of participant experiences of each therapeutic session was administered weekly throughout the intervention to assess the relationship between the therapist and the young person. This is widely used in children and young people, NHS Talking Therapies services, to assess routine outcomes and has shown good psychometric properties (Duncan et al. 2003). Scores are calculated by summing the scores across the four items; a score below 36 total or nine on any subscale is suggested as a source for concerns.

*Recovery Questionnaire (ReQuest‐YP; John et al*. 2015 *)*: Examines recovery of functionality and outlook posttreatment. Evaluation of the ReQuest‐YP demonstrated good internal consistency in a sample of young people in the community (α = 0.95; John et al. 2015) and good internal consistency and test–retest reliability in inpatient services (α = 0.91; ICC = 0.91; Bentley, Bucci, and Hartley 2019).

*University of Rhode Island Change Assessment Scale (URICA)*: Examines a person's motivation to change in relation to a specific ‘problem’. Originally developed for working with addiction, it is a 32‐item self‐report measure providing an overall readiness to change score and four subscale scores related to stage of change: precontemplation, contemplation, action and maintenance (DiClemente, Schlundt, and Gemmell 2004; McConnaughy, Prochaska, and Velicer 1983). The psychotherapy version has demonstrated good internal reliability (α = 0.75–0.83 across subscales; Pantalon et al. 2002).

### 2.4 Procedure

After individuals were referred by clinicians within ED services across four NHS Trusts, their eligibility was checked via telephone call. If the young person met the criteria and maintained interest in participating, a research assessment was arranged in which consent was taken. This was largely completed face‐to‐face, except for one participant who completed the baseline assessment online via video call with consent being audio‐recorded. For participants under 16, caregivers were also required to give consent. Baseline assessment measures were completed immediately following consent.

Participants then completed the RIDE reformulation over five sessions, taking place approximately once a week, each lasting approximately 50 min. Two measures (YP‐CORE and SRS) were completed weekly, either face‐to‐face in the appointment or via an online survey platform. As soon as possible after the final therapy session, participants were asked to complete a final research appointment where they completed the postintervention outcome measures. A further follow‐up took place 2 weeks after the postintervention assessment. Participants were reimbursed with a £30 shopping voucher for their participation.

#### 2.4.1 RIDE

Researchers followed the RIDE treatment guide. RIDE is adapted from previous CAT‐informed reformulation interventions developed by clinicians and examined in a case series study (Haw et al. 2024). A guidance document is available in Data S1. RIDE focused on broader underlying, precipitating and maintaining relational patterns rather than specific ED symptoms. Informed by principles of CAT, sessions one to four focused on ‘mapping’ to form a collaborative understanding of the young person's relational and coping patterns (reformulation), in a ‘map’ (Sequential Diagrammatic Reformulation; SDR). Ways to move away from unhelpful patterns, referred to as ‘exits’, were noted for the young person to explore following the intervention. A verbal summary or ‘goodbye’ letter that captured the reformulation was shared with the young person. The aim of the final session was as a consultation session to consolidate the understanding developed and share this with other key individuals (family and mental health professionals). If an individual did not want to share the reformulation with others, the final session was used as an opportunity to reflect on what needs to change in their system to allow safe and useful sharing and work together in the future.

The intervention was delivered by two members of the research team (CG and GM). To reduce the potential bias, neither therapist held the dual role of therapist‐researcher (i.e., acting as therapist and also completing assessments with participants) with any individual participant. The therapists were both white, female, aged final year trainee clinical psychologists. Both researchers were familiar with CAT and had experience working therapeutically with young people and their families. Researchers each completed approximately 17 h of training with CAT‐qualified practitioners prior to delivering the intervention and received clinical supervision from a qualified CAT practitioner and supervisor throughout the intervention. Additional ad hoc clinical supervisory support was received from senior clinical psychologists within the research team.

### 2.5 Data Analysis

To evaluate the primary aims of acceptability and feasibility, descriptive statistics of recruitment, retention and attendance rates were generated. Data completeness was evaluated by summarising missing data rates. The secondary aim involved exploration of clinical change. Meaningful data was explored, and trends over time were examined for all secondary measures. Means, standard deviations and confidence intervals were calculated and analysed using IBM SPSS Software (Version 29; IBM Corp 2022) to determine pre/postassessment treatment mean changes. Clinically significant change was also calculated for psychological distress, ED severity and personal recovery using the Reliable Change Index (Jacobson and Truax 1991). A Jacobson–Truax plot has been created using the rciplot package (Hagspiel 2023) in R (R Core Team 2021).

## 3 Results

### 3.1 Participant Characteristics

Participant ages ranged from 15 to 24 (mean: 20.25, *SD* = 3.58). All participants were white, and the majority identified as female. Demographic information is presented in Table 2. One participant did not know their specific ED diagnosis and it was not reported by the referring clinician. Data S2 contained item‐level results from the EDE‐Q.

**TABLE 2:** Demographic information at baseline.

Characteristics	*n*
Gender	
Female	7
Male	1
Ethnicity	
White	8
Reported eating disorder diagnoses	
AN	3
EDNOS	3
BED	1
Unknown	1
Previous psychological therapy	
Cognitive behavioural therapy	3
MANTRA	1
Counselling	1
None	3
Additional mental health difficulties	
Depression/low mood	4
Anxiety disorders and phobias	5
None	1
Psychiatric medication	
Antidepressant	4
Mood stabiliser	1
None	3

Abbreviations: AN, anorexia nervosa; BED, binge eating disorder; BN, bulimia nervosa; EDNOS, eating disorder not otherwise specified; MANTRA, Maudsley model of anorexia nervosa treatment for adults (Schmidt, Wade, and Treasure 2014).

### 3.2 Feasibility and Acceptability

#### 3.2.1 Referral Rates

**FIGURE 1:**
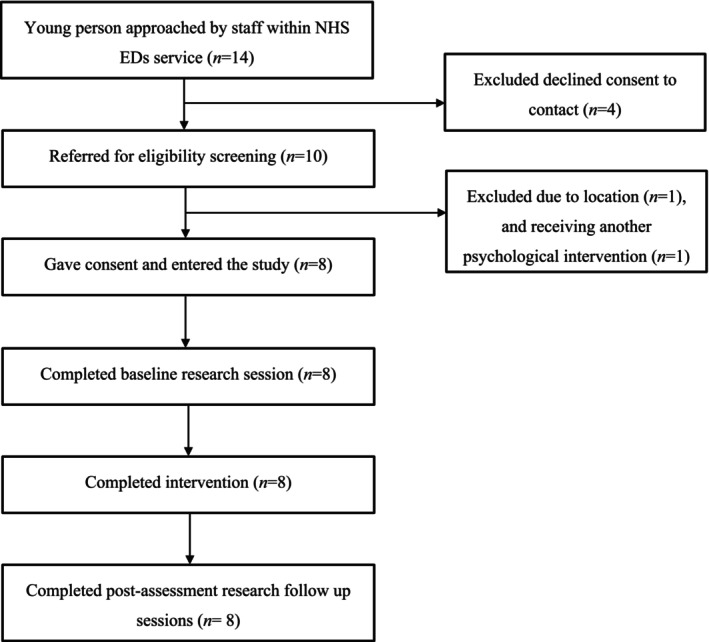
Consort diagram of participants per referral stage. Total numbers of participants were recorded at each stage of the study. Four known young people refused consent to be contacted by the researchers. All eligible referred participants consented to take part, completed all intervention and research sessions.

The CONSORT diagram in Figure 1 shows the sample size at each study stage. Clinicians reported that four young people approached to participate did not consent to be contacted by the researchers. Feedback suggested reasons for declining referral were as follows: concerns about meeting with other people (i.e., due to social anxiety), concerns about completion of questionnaires, time constraints or other commitments or concerns about the stage of their ED journey and building a therapeutic relationship for only 5 weeks. Ten young people gave consent to be contacted by the researchers. Two of the 10 people referred to the project did not meet eligibility criteria and were excluded at the screening stage. One was excluded due to living out of the country and one was excluded as they had been allocated to another psychological intervention within the NHS service since referral.

#### 3.2.2 Retention to Treatment

All eight remaining young people gave consent to participate and completed the baseline (pretherapy) assessment. Of those who consented to participate, everyone (*n* = 8) attended all five sessions.

#### 3.2.3 Clinical Outcome Data Completion

Baseline and postintervention measures were completed by all eight participants. Weekly outcomes (YP‐CORE, SRS) were completed 100% of the time. Notably, data completeness was 100% for all measures apart from YP‐CORE weekly outcomes where one item was missed on one occasion (99.75%).

#### 3.2.4 Acceptability

All participants (*n* = 8) felt it was ‘certainly true’ that they were treated well by their therapist; their views and worries were taken seriously, and they were given enough explanation about the therapy being offered at the start. Most participants (*n* = 7) felt that it was ‘certainly true’ that they felt listened to in therapy sessions. Three quarters felt it was ‘certainly true’ (*n* = 6) that it was easy to talk in therapy sessions and the therapist knew how to help them. Finally, over half of participants (*n* = 5) felt it was ‘certainly true’ that they would suggest this therapy to a friend in need of this sort of help. One participant indicated they ‘did not know’ if they would recommend this sort of therapy to a friend in need of psychological help when asked. No other responses were lower than ‘partly true’.

Participants were provided space to detail what they liked and what they felt could be improved about the intervention, as well as additional comments. All participants identified positive aspects of the intervention. Positive intervention outcomes, including gaining better recognition and understanding of their difficulties and being kinder to themselves, were identified by most participants (*n = 7*):

[The therapist] was very open to allow me to talk about many aspects of my life which was useful to understand those patterns of thinking more

The therapy helped me recognise my problems and gain a better understanding

Two participants specifically referred to the intervention feeling collaborative. Therapeutic qualities, including feeling listened to and understood in a nonjudgemental manner, were commonly identified (*n =* 6):

I felt very supported and listened to

I felt there was no judgement and that we collectively wanted to find coping mechanisms and better ways to cope

The visual process of mapping (SDR) was described positively by four participants:

The use of mind maps helped me to understand how my feelings and actions were connected so it gave me a better understanding of what's going on inside my head

The mapping out made things I find it hard to talk about easier to think about cause I could visualise it. The way we made the links on the paper together made it feel like a collaboration

Other feedback included positive reflections on the person‐centred nature of the intervention (*n* = 1), pace of the intervention not feeling rushed (*n* = 2) and intervention increasing commitment and hope to further therapy (*n* = 2):

I also really appreciated that the therapy felt quite natural and not too regimented or ‘one size fits all’ … I really felt cared for in the space and didn't feel rushed or pressured

This was my first positive experience of therapy. If it hadn't been with [the therapist] and I hadn't felt comfortable (which I don't usually) I would have refused future treatments as that was my plan that it just wouldn't work for me. This therapy has made me want to keep trying

When asked what they disliked or would improve about the intervention, four participants did not identify anything. One participant disliked practical aspects of the therapeutic space, specifically a lack of tissues. The main area of feedback focused on intervention length and the amount possible to cover during intervention (*n* = 3). While some participants appreciated that intervention did not feel rushed, two felt RIDE should have involved a greater focus on ‘exits’, and another felt the therapy ended as they were starting benefit. One participant felt intervention should involve further check‐in sessions due to sensitive topics discussed and feelings evoked:

Because of how short the trial is and how vulnerable I feel coming out of it I'm not sure I'd recommend it unless someone was going straight into therapy (like me) or there were some kind of follow‐up wellbeing check‐ins or sessions. More focus on practically tackling specific eating behaviours if someone is in crisis

### 3.3 Clinical Outcome Measures

As detailed, data from one item on one measure (YP‐CORE) was missing, and as outlined in the measure's manual, the prorated mean was utilised. Summary statistics for all secondary outcomes are presented in Table 3. All outcomes improved when comparing baseline to postintervention. Mean differences suggest trends towards improvements in psychological distress (YP‐CORE), eating disorder difficulties (EDE‐Q), recovery (ReQuest‐YP) and readiness to change (URICA). The smallest change was observed on the contemplation subscale of the URICA suggesting this remained relatively consistent across intervention.

**TABLE 3:** Clinical outcome data.

Variable	Baseline mean (SD)	Postintervention mean (SD)	Follow‐up mean (SD)	Postintervention mean difference (95%CI)	Follow‐up mean difference (95%CI)
YP‐CORE	19.13 (6.29)	12.75 (6.04)	13.50 (5.26)	−6.38 (−8.74, −4.01)	−5.63 (−7.67, −3.58)
ReQuest‐YP	43.50 (12.08)	46.63 (9.59)	47.00 (10.50)	3.13 (−2.69, 8.94)	3.5 (−4.78, 11.78)
URICA readiness for change	9.95 (2.07)	10.61 (1.00)	10.25 (1.36)	0.66 (−0.82, 2.14)	0.30 (−0.84, 1.45)
URICA subscales					
Precontemplation	10.50 (2.20)	9.25 (1.98)	10.38 (2.56)	−1.25 (−3.88, 1.38)	−0.13 (−1.57, 1.32)
Contemplation	29.25 (4.43)	29.50 (2.51)	28.75 (3.06)	0.25 (−2.87, 3.37)	−0.50 (−3.11, 2.11)
Action	25.63 (6.26)	27.63 (4.60)	27.63 (4.27)	2.00 (−1.46, 5.46)	2.00 (−0.90, 4.90)
Maintenance	25.38 (4.24)	26.63 (2.33)	26.13 (1.96)	1.25 (−2.20, 4.70)	0.75 (−3.19, 4.69)
EDE‐Q total	4.05 (1.47)	3.40 (1.58)	*n/a*	−0.65 (−0.92, −0.38)	*n/a*
EDE‐Q subscales					
Restraint	4.19 (0.79)	3.30 (1.72)	*n/a*	−0.89 (−2.17, 0.39)	*n/a*
Eating concern	3.43 (1.72)	2.68 (1.18)	*n/a*	−0.75 (−1.21, −0.19)	*n/a*
Shape concern	4.28 (2.08)	3.92 (1.94)	*n/a*	−0.36 (−0.68, −0.05)	*n/a*
Weight concern	4.30 (1.84)	3.70 (1.76)	*n/a*	−0.60 (−1.14, −0.06)	*n/a*

Abbreviations: CI, confidence interval; EDE‐Q, Eating Disorders Examination Questionnaire; n/a, not applicable; ReQuest‐YP, Recovery Questionnaire for Young People; URICA, University of Rhode Island Change Assessment Scale; YP‐CORE: Young Person's Clinical Outcomes in Routine Evaluation.

Most outcomes also demonstrated sustained improvement from baseline to two‐week follow‐up. Positive trends in psychological distress (YP‐CORE), recovery (ReQuest‐YP) and readiness to change (URICA) were maintained at follow‐up. With regards, the URICA subscales exploring the stages of change, mean differences suggest contemplation decreased, while action and maintenance increased at follow‐up.

#### 3.3.1 Reliable Change

**FIGURE 2:**
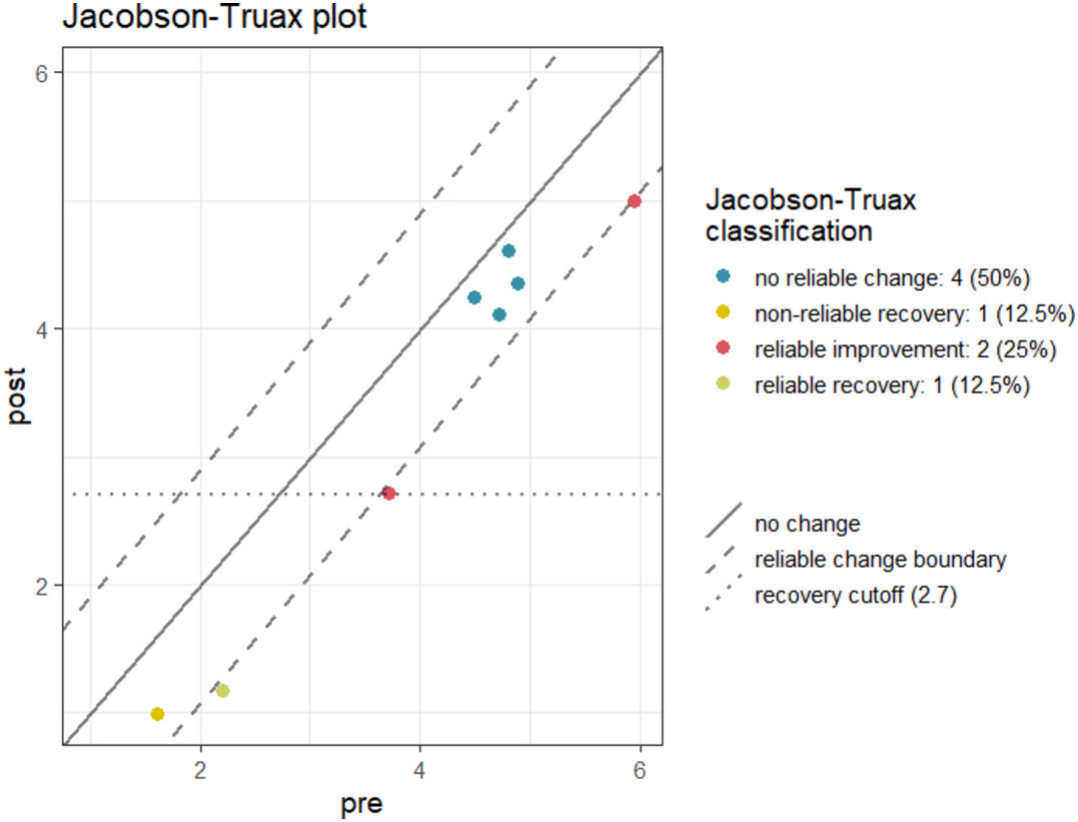
Jacobson plot for the EDE‐Q. The Eating Disorder Examination Questionnaire (EDE‐Q; Fairburn and Beglin 1994) was administered pre and postintervention. A Jacobson plot shows reliable change for participants across intervention. One participant demonstrated non‐reliable recovery across intervention. Three people showed reliable improvement in terms of eating disorder severity across the intervention. Of the three participants who demonstrated reliable improvement, utilising the clinical cut‐off 2.7, only one participant demonstrated reliable recovery across the intervention. This participant was already in the nonclinical range pretherapy.

For the YP‐CORE, EDE‐Q and ReQuest‐YP, posttreatment reliable change was calculated using the reliable change index (RCI; Jacobson and Truax 1991) and Cronbach alphas presented in the method section. Due to a lack of comparable studies reporting coefficient alphas, reliable change could not be calculated for the URICA. Reliable improvement was observed for three people on psychological distress (YP‐CORE) and three people on ED severity (EDE‐Q; see Figure 2). However, of the three participants who demonstrated reliable improvement with regard to ED severity, utilising the clinical cut‐off 2.7, only one participant demonstrated reliable recovery across the intervention. Notably, this participant was already in the non‐clinical range pre‐intervention. No participants showed reliable change with regard to personal recovery (ReQuest‐YP). No participants showed a reliable deterioration across any domains.

#### 3.3.2 Sessional Measures

**FIGURE 3:**
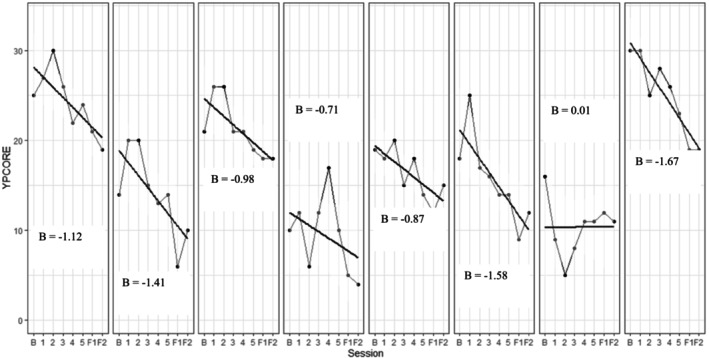
Graph of change in psychological distress (YP‐CORE) over study involvement for each individual participant. The Young Person's Clinical Outcome in Routine Evaluation (YP‐CORE; Twigg et al. 2009) was administered at baseline, following all intervention sessions, post‐intervention (F1 on graph) and follow‐up to measure psychological distress (F2 on graph). Results were plotted on individual graphs to explore change for each individual participant. Some participants demonstrated an initial increase in distress during the intervention. All participants showed reductions in distress across intervention.

**FIGURE 4:**
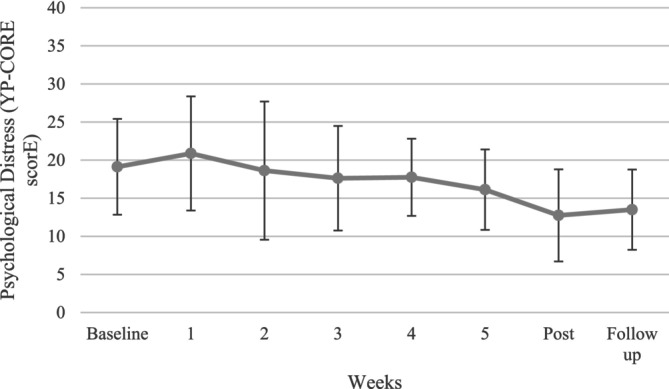
Graph of average group change in psychological distress (YP‐CORE) over study involvement. The Young Person's Clinical Outcome in Routine Evaluation (YP‐CORE; Twigg et al. 2009) administered at baseline, following all intervention sessions, postintervention and follow‐up to measure psychological distress. Average group changes were plotted to explore overall group changes across intervention. Whiskers represent standard deviation around the mean. Overall trends suggest a reduction in psychological distress across intervention.

Psychological distress was assessed on a sessional basis throughout the intervention using the YP‐CORE. As shown in Figure 3, there was variation in starting reported distress levels between participants, but all participants demonstrated a reduction in distress across intervention. Fluctuation in distress also varied considerably between participants. The majority (*n* = 6) experienced some increase in distress during intervention, which subsequently declined to show improvement over time. One participant showed an initial sharp decline in reported distress, which then stabilised over intervention. The average group change on the YP‐CORE is reported in Figure 4.

Weekly SRS scores were also collected to assess therapeutic alliance across intervention. Most participants consistently scored above the cut off for concern (36) across intervention (M = 38.13, range 22–40). Several participants (*n* = 4) indicated improved therapeutic alliance across intervention, with one starting just below cut off (34). One participant reported scores significantly below the cut off for concerns in the beginning of intervention (week 1 = 22, week 2 = 24), which rose throughout, but ended on the cut off at the end of the intervention.

## 4 Discussion

This study aimed to assess the feasibility and acceptability of a brief, CAT‐informed reformulation (RIDE). Findings suggest that RIDE is feasible and acceptable to young people experiencing ED. This is evidenced by the high participation rate of eligible referred individuals (100%), excellent retention to treatment (100%) and high rates of outcome measure completion. High levels of satisfaction with the intervention were reported by participants. All felt they were treated well by their therapist; their views and worries were taken seriously, and they had a good understanding of the intervention at the start. Participants largely felt listened to by their therapist, whom they felt knew how to help them and found it easy to talk within the sessions. Many would recommend this type of therapy to a friend struggling with similar difficulties. A small number of participants offered ways they felt the intervention could be improved.

Following treatment, improvement across most outcome measures was observed. Greatest reductions were observed for psychological distress (YP‐CORE), which were maintained at follow‐up, and for the range and severity of ED symptoms (EDE‐Q). The biggest reduction in ED‐related symptomology was observed on the eating concerns subscale. Small improvements were maintained for recovery (ReQuest‐YP) and readiness to change (URICA) at follow‐up. In terms of stages of change, small improvements on the action subscale were maintained at follow‐up, while precontemplation, contemplation and maintenance remained consistent or decreased to below small effect sizes at follow‐up. Preliminary results show promising benefits across clinical outcomes but should be interpreted with caution; the small sample size limits the generalisability of findings. As this was a case series design and lacked a comparison group and randomisation, alternative explanations (e.g., regression towards the mean and ongoing support from services) for improvement of scores cannot be eliminated, and changes cannot be confidently attributed to intervention. Further controlled trials are required to determine if improvements are the result of the intervention and are replicable on a larger scale.

Some concerns raised by those who declined referral (*n* = 4) may be addressed if delivered within services, for example, concerns around meeting further clinicians outside of their clinical team. Of those who consented to treatment, high levels of engagement and adherence to treatment were particularly promising with regard to the acceptability and feasibility of the intervention. High dropout rates can be a significant problem in EDs treatment, with estimates varying between 20% and 40% dropout in outpatient treatment (DeJong, Broadbent, and Schmidt 2012; Waller et al. 2009). This study adds to the growing evidence of low dropout in CAT interventions comparative to other psychological interventions (Simmonds‐Buckley et al. 2022); however, results should be examined cautiously. The brevity of intervention reduced opportunity for dropout, and results may reflect long wait times for other ED treatment and desire for treatment within the small sample (Iacobucci 2021; Nuffield Trust 2023).

Three participants commented on the length of intervention; one specifically suggested offering follow‐up sessions, with two wanting a greater focus on ‘exits’ from existing patterns. The current manual emphasised building an understanding of the young person's difficulties through SDR. Though some feedback suggested the intervention pace and time offered to understand wider difficulties in relation to ED behaviours was appropriate, it is important to consider individual needs in intervention length. An aim of feasibility and acceptability studies is to identify improvements to novel interventions to avoid difficulties arising at a larger scale evaluation. Eight‐session CAT for anxiety and depression, where there is clinical complexity, demonstrated promising practice‐based results similar to CBT (Wakefield et al. 2021). While remaining ‘brief’, extending to an eight‐session protocol would offer greater space for recognition and revision. At present, eight‐session CAT‐informed interventions have not been explored in ED treatment among young people. A further suggested option is offering follow‐up sessions, common in CAT practice, as further space to reflect and consolidate. As endings are an important experience within CAT therapy (Ryle and Kerr 2020), future research should explore the impact of endings within shorter CAT interventions.

This study has implications for clinical practice and future research. However, methodological limitations must be considered. The lack of randomisation or control group comparison means outcomes cannot be conclusively attributed to treatment. The small sample size means effect size estimates should be interpreted cautiously. For example, the study is likely to under or overestimate treatment effects comparative to what would be seen in a larger RCT (Richy et al. 2004). The sample also lacked demographic diversity; all participants were white and identified as cisgender; yet transgender and gender‐diverse populations are at increased risk of EDs (Simone et al. 2022). It remains unclear whether a brief CAT‐informed reformulation intervention would be acceptable within marginalised communities who may face additional barriers in accessing effective treatments (e.g., lack of gender‐affirmative care and lack of consideration of cultural differences on symptoms; Hartman‐Munick et al. 2021; Griffiths 2023). It is also notable that five participants had previous experiences of therapy. These results are consistent with wider research that relapse rates for ED can be high (Khalsa et al. 2017), resulting in recurrent service contact. While the fact that these young people continued to struggle with EDs and were seeking further intervention supports the need for alternative treatments, it is not clear how these previous experiences impacted the results. In the future, it would be beneficial to examine the intervention with more people with no prior therapy experience. We designed RIDE as an intervention that could be delivered when a young person is first referred into an ED service. Based on these results, we see that this population will often include young people with previous service contact. RIDE may provide a useful way of understanding why previous interactions with services have not resolved their difficulties or how these have reoccurred.

A further limitation exists within intervention delivery, despite possessing substantial therapeutic experience and training in CAT, the study therapists were not CAT‐accredited practitioners. Due to funding and time restraints, the quality of CAT session delivery was also not rigorously evaluated, for example, using a measure of therapist competence in cognitive analytic therapy (CCAT; Bennett and Parry 2004). Therefore, fidelity to the CAT model and RIDE manual cannot be assured. Consistent and close supervision by a CAT‐accredited clinical psychologist does, however, mitigate against this risk.

In the UK, typical divisions between CAMHS and those services provided to adults can create barriers and delays in accessing treatment for those nearing transition age (> 18; Allen et al. 2023). Recent years have seen convincing arguments for services spanning this age divide (McGorry et al. 2022). First Episode Rapid Early Intervention for Eating Disorders (FREED) services spanning 16‐ to 25‐year‐olds have been piloted with positive outcomes (NHS England 2020; Austin et al. 2022; Fukutomi et al. 2020). Outcomes suggest the current intervention is acceptable to young people across this age range (15–24 years) and may be well suited to such services with a focus on early engagement (Allen et al. 2020), where a CAT‐informed assessment and formulation may improve motivation to change and early symptom change. Aligned to CAT aims (Ryle and Kerr 2020), positive feedback cited increased understanding of difficulties, often facilitated by SDRs. Improved understanding may be a catalyst for improved motivation and engagement with treatment in a population often categorised as ambivalent to change. However, clinician, service and system‐level factors can also impact the duration of untreated ED; for example, long waitlists or services adopting a ‘watch and wait’ approach if someone is not deemed “unwell enough” (Allen et al. 2023). Yet a longer duration of untreated EDs can worsen outcomes (Fernández‐Aranda et al. 2021). RIDE may be an accessible and beneficial early intervention in such cases. Further research should explore brief transdiagnostic CAT‐informed reformulation intervention for people presenting with ED‐related concerns but who may meet full diagnostic criteria.

Despite limitations, this case series shows promising results on the feasibility and acceptability of a brief CAT‐informed reformulation intervention for EDs among young people. Whilst positive change has been indicated, results should be interpreted with caution. This study is the first step in exploring a novel CAT‐informed reformulation intervention and results warrant further investigation on a larger scale and with scientifically rigorous research designs, likely a feasibility RCT followed by a full efficacy RCT if further indicated.

## Author Contributions

C.G, G.M., P.T., S.H. and B.D. designed the study. I.G. was involved in study recruitment and management. C.G. and G.M. collected the clinical data and delivered the intervention. C.G. analysed the data and drafted the manuscript. All authors contributed to manuscript writing and editing and approved the manuscript for submission.

## Ethics Statement

This research was granted approval from the University of Manchester Sponsorship panel and the NHS Research Ethics Committee (REC reference: 23/YH/008). Recruitment took place within four local NHS trusts who also issued local approval. All participants gave informed consent.

## Conflicts of Interest

The authors declare no conflicts of interest.

## Supporting information


**Data S1** Supporting information.

**Data S2** Supporting information.

## Data Availability

The study data will be available upon reasonable request.
