# Supplementary material for: Brief Cognitive Analytic Therapy (CAT)‐Informed Reformulation for Young People With Eating Disorders: A Case Series
Source: Clin Psychol Psychother. 2025 Apr 2;32(2):e70043. doi: 10.1002/cpp.70043 (PMC11963221; doi:10.1002/cpp.70043)
Supplement: Supplementary file 1 — Data S1 Supporting information. [file CPP-32-e70043-s001.docx]

**Brief Cognitive Analytic Therapy for Young people in Eating Disorder Services**

**GUIDANCE DOCUMENT**

**Peter Taylor**

**Clive Turpin**

**Samantha Hartley**

**Ian Gill**

The initial CATCH intervention was largely based upon: Sheard, T. , Evans, J. , Cash, D. , Hicks, J. , King, A. , Morgan, N. , Nereli, B. , Porter, I. , Rees, H. , Sandford, J. , Slinn, R. , Sunder, K. and Ryle, A. (2000), A CAT‐derived one to three session intervention for repeated deliberate self‐harm: A description of the model and initial experience of trainee psychiatrists in using it. British Journal of Medical Psychology, 73: 179-196. doi:[10.1348/000711200160417](https://doi.org/10.1348/000711200160417)

The approach was adapted initially to 1) shift the focus from overdoses to self-harm more broadly, 2) move away from a hospital based context for the intervention, 3) reduce the number of sessions to two.

In revising the intervention for young people (CATCH-Y), we sought the views of young people who have utilised Child and Adolescent Mental Health Services (for difficulties including self-harm) in both the community and inpatient settings. We also consulted clinicians who work in this area. The main changes were: 1) Increase of session number to 5, with the 5^th^ session incorporating a handover to coordinating clinician and/ or family member; 2) Reduction in standard session length to 60 minutes and flexibility in this; 3) To include explicit provision of psychoeducation where appropriate; 4) Flexibility in the location of sessions; 4) Emphasis on systematic factors/ opportunities as part of the reformulation and potential exits (e.g. family-based treatment/ social care involvement).

The terms ‘young person’ and ‘client’ are used interchangeably throughout this document.

Version 2 of this document was developed as a result of a case series of the CATCH-Y model undertaken by Rebecca Haw and Molly Marsden (trainee clinical psychologists at the University of Manchester), under the supervision of the main authors. This study is reported in full elsewhere: (Haw et al., 2022; doctoral thesis).

The guidance has recently been adapted for use with young people in eating disorder services, as part of work with on the RIDE (Relational Interventions for Difficulties with Eating) project.

**OVERVIEW**

This guidance document gives a brief overview of a five-session (four plus one) Cognitive Analytic Therapy (CAT) intervention aimed at young people (aged 14-18) in eating disorder services. This guidance document assumes an existing knowledge of CAT and does not provide a detailed definition of CAT concepts and ideas. CAT is a personalised and idiosyncratic therapy that is guided by the reformulation and therapeutic relationship created between therapist and client. Therefore, the document offers guidance to core elements of consideration, rather than a ‘how-to’ or ‘step-by-step guide’. Practitioners using this approach should already have a good grounding in CAT, experience of working with young people in a clinical capacity and appropriate clinical supervision structures in place to support the dynamic and formulation-driven adaptation and implementation of the guidance.

The intervention is based around five face-to-face sessions, preferably spaced a week apart. The intervention centres on developing a shared, collaborative, relational understanding of a client’s experiences, drawing upon the Cognitive Analytic Therapy (CAT) framework for making sense of these experiences. Broadly the goals of the intervention are to:

- Develop a shared relational, parsimonious understanding of the client’s experience of emotions, relationships, coping, and specific behaviours (including but not limited to those related to eating difficulties), capturing the antecedents, consequences, patterns, and -crucially – the understandable functions related to this behaviour.
- Using CAT constructs of ‘Reciprocal Roles’ and ‘Procedures’ (see below) to help develop clients’ awareness and understanding of these experiences. These concepts do not necessarily need to be named in the therapy but should be used where appropriate by the therapist to help explore, develop and elaborate on the client’s understanding of their experiences, bringing a compassionate and relational lens to this.
- Where relevant, explore an understanding of the young person’s ‘relationship to help’, experience of services and motivation to change
- Where relevant, highlight common relational processes (e.g. seeking control, striving, emotional numbing etc.) between different types of observable or internal experiences (e.g. eating restriction, self-harm, etc)
- Where appropriate, provide an initial exploration of how a client might in future and with support use their developed awareness to make any changes they might want to, in line with their ZPD and motivation to change
- Roles, procedures and exits should include explicit consideration of systemic experiences, influences and opportunities, such as relationships with systems in the young person’s life (e.g. school or mental health services) and potential options for service-level exits (e.g. liaison with social care), alongside the individual young person’s active role.
- Share this understanding with the young person’s coordinating clinician and/ or family members to handover this understanding in the hope it will engender greater relational understanding of their experiences, inform care planning and highlight and/or avoid any potential blocks to effective care or potential iatrogenic harm.

**Introducing the Intervention**

As this is a short intervention it is necessary to be mindful of clients’ expectations about the intervention and transparent about the aims, potential benefits, and possible risks. It is important to be clear about the length of the sessions and the intervention from the start, and may be helpful to remind clients of this as work progresses and use the brevity to aid focus.

Clients will be made aware at the baseline assessment that the intervention is part of a research study. If directly asked about the impact/helpfulness of the intervention, it can be stated that you are hoping to find out whether this sort of brief intervention can be helpful for people with eating and other difficulties, and that you know that many people appear to value and benefit from this sort of intervention (i.e. CAT and CAT informed approaches), although cannot give assurance.

When introducing the therapy it could be suggested that the goal of the intervention is to develop a way of understanding the person’s experiences, that looks at the patterns that people can get stuck in, rather than necessarily coming up with solutions or new ways to cope. The intervention can be framed as wanting to get ‘underneath’ or ‘behind’ the surface levels symptoms, problems or risks and understand the themes, patterns or processes that are driving these. The emphasis is on understanding why experiences are present in a historical and current relational context –and not to provide a ‘quick-fix’ solution to making changes; acknowledging that other aspects of service provision might currently be requiring change (such as meal plans etc) that is unwelcome to the young person (without disparaging these). The intervention could be introduced as an opportunity to think about these experiences and highlight how developing an understanding of them could support future change.

The content of therapy should be briefly discussed; i.e. it will involve discussing experiences and ideas, and drawing out patterns that occur in their relationship with others and themselves. Emphasis should be placed on doing what works for that young person and figuring that out together during therapy- i.e. in the relative balance of mapping and talking, questions and hypotheses. At this point the therapist might explore previous experiences of mental health in general and what has felt helpful/ unhelpful.

***T:*** *So in these sessions we have together, a big part of it will be talking about your experiences, including things you might struggle with or find difficult, and together we will try to better understand these experiences and difficulties. We know that you experience difficulties with [XYZ] – we want to get underneath those experiences to understand the patterns or themes that might link them together or drive them so we know what might be helpful and so things might be less confusing. To do this, we’ll talk about how things are for you, your feelings and relationships. Sometimes this might be hard to do – we’ll check in together to make sure we go at a pace that’s ok with you and we can use different ways of developing an understanding together- like talking, or drawing or writing or even moving around.*

**Therapist Style**

In line with a standard CAT approach the therapist should work with the following therapeutic principles:

- Working collaboratively, getting alongside the client to try and understand their world and their experiences.
- Seeking understanding of the whole person (in a parsimonious way) rather than a focus on diagnostic constructs or symptoms
- Being aware of the inherent power imbalance between therapist and client but also between adult and child, and working with an awareness and mitigation of its impact.
- Being curious, open minded and compassionate.
- Showing appropriate empathy and concern (avoiding alarmist or judging comments).
- Within CAT therapists can be proactive, making suggestions or suggesting hypotheses, sharing their thoughts. However, this should be carefully paced in light of the client can take in, to avoid running ahead of them or leaving them feeling overwhelmed or pressured to respond in a certain way.
- Particular care should be taken to work within a young person’s Zone of Proximal Development (ZPD) in terms of their emotional and relational literacy. Our work has indicated that young people who finds discussing their emotions or relationships tricky will also likely find this tricky in CAT. This doesn’t necessarily preclude this approach but will necessarily have implications for the nature of the conversation, pace of joint formulation and potential positive impact of the intervention.
- Making decisions collaborative and explicit, drawing on ‘in the room’ experiences and relational encounters, where this is tolerable.
- “Push where it moves” – try to identify where new understanding or change might be possible for that specific client and focus on those areas.
- Be mindful of the enactments (e.g. a pull to act or respond in a particular way to a client) that you may be drawn into and try not to “join the dance”, or to be aware when you may have already been drawn into this.

**Suitability and service context**

Reflections from our previous studies indicate that it is helpful to think with young people and clinicians about when and how to make the best use of this type of intervention, using CAT concepts of ZPD and ‘pushing where it moves’. Repertory grids, which have been used in both clinical practice and research evaluations, might be a useful triage tool as they require similar processes as CAT. Clients who are more able to verbalise (in any terms) their emotional and relational experiences might get more out of the therapy. This would not preclude others taking part but might help with decision making and collaborative development of expectations. It is also important to consider the wider system here- the relationships between the client and the clinicians, where the therapists sits in this. Looking ahead to session five, these relationships will influence how safe and effective the sharing of information and relational understanding is for the client. In the context of ED services specifically, this intervention will likely sit alongside others, such as family based treatment or family therapy, dietetic input and care coordination. It’s important to view the CAT-informed work as potentially feeding in to these other aspects of care provision.

**Session structure**

Most therapeutic approaches, including CAT, place value on the ‘therapeutic frame’. Alongside this, young people value flexibility and adaptability in terms of session location and length. It is therefore important that the therapist balances these competing demands and takes time to explore with the young person; a) where therapy would be best located (default is at a clinic location but options might include school, home or GP surgery) and b) how long therapy lasts (maximum is 60 minutes per session but a minimum of 30 minutes is acceptable). Exploration and contracting around these elements should occur prior to session one and be finalised there. Within the following sessions the agreement about location of the sessions should be adhered to, but the length of the session might be increased (e.g. from 30 to 60 minutes) if re-contracted in the previous session.

**Session one and an overview of key therapeutic processes**

The initial session should last around 60 minutes (though see above), and include:

- Provide a brief introduction to what the therapy involves (see above), including the number and duration and focus of the conversation, checking how this sounds to the client and fits with their expectations. The therapist should highlight how to aim of this intervention is not to jump to changing thoughts and feelings related to weight, shape and eating but to understand the young person’s varied and subjective experiences – how they impact, their function, their relational patterns etc, with the aim of enhancing understanding and identifying what might help (now or in future). (5 minutes)
- Reiterate requirements around risk and confidentiality (this will have been covered in their previous meeting with the researcher) including briefly referring back to the plan discussed in their first meeting about what might be done if there is a concern about risk to themselves or others. (2 minutes)
- Exploration of sharing information with parents in line with the young person’s age and competence. Where competent, the young person can decide for no information from therapy to be shared with parents (aside from that pertaining to risk, which would be done by the care coordinator).

***T:*** *So, we have talked about how you are with this service, because of worries around eating and how you feel about your weight. In this therapy we might talk about those things, but we might also talk about more general stuff. So things like how you feel about other people in your life, and how you feel about yourself. It’s like we want to understand what might link different aspects of your experiences together - what the themes might be or how to make sense of it all and what might be helpful.*

**Adapted Psychotherapy file and other tools**

Participants can be sent the Adapted Psychotherapy File (see Appendix I) by post or email following the initial assessment, to complete before the first therapy session. If clients have not completed this it can be done at the start of the session. If completed in the session this can be done in an interactive manner, asking the client the questions verbally, with the file visible to both therapist and client. It is not essential to complete the File. It may be that in starting a session there is already important content to pick up on and that going through the File would only disrupt this process. However, if the File has been offered (whether completed or not) then it should always be discussed (otherwise the client may feel they are being asked to do something which is not important). As with any ‘tools’ used in CAT, the collaborative review should utilise reflections on both process and content.

If the File is completed then the client’s responses on the File should be discussed. The goal of this activity is not to collect data or get to a “correct” answer, but to open a discussion about the client’s experiences. It should be explained that the file is not an exhaustive list and won’t fit for everyone.

The therapist should explore with the client if any of the feelings or patterns covered in the File seem particularly relevant to their experiences. Where this is the case, it can provide a potential starting point in mapping out the client’s experiences. For example, the therapist can start this process by writing out the states/feelings on a separate sheet of paper.

Where feelings or patterns listed in the File have some relevance, but do not seem to capture the client’s experience fully, this is an opportunity to try to further elaborate on the client’s own experience (e.g., “So the feeling is not quite like X, how would you say it is different? Is it more like …”). This would be another starting point for formulation.

If clients struggle to engage with the File or identify any feelings or patterns that fit for them, it is important to reflect that this is fine, the ideas in the File will not fit for many people (while noting as a therapist any implications for possible relational hypotheses). This is then a starting point to suggest working together to try and better understand the client’s own experiences. If it feels acceptable within the therapy relationship, exploration of what the barriers to completing the File were can be helpful, as this may reveal important relational patterns (or provide hints of possible enactments of such patterns) and associated feelings. For example, a concern about “getting it wrong” or “trying to get it just right” when filling in the File may hint at a tendency to try and please or at an underlying perfectionism.

**Mapping – getting started**

A young person might find it difficult to express experiences in response to open questions or those targeted at relational, emotional or problematic experiences. In this context, additional tools can be employed. The 24 hour clock technique involves asking a young person to recount the previous day, hour by hour starting when they get up (a visual aid could be useful here). The therapist should ask the young person to describe what happens in details – events, people, experiences, reactions, responses, interactions. The therapist should adopt and, curious and interested stance and listen out for potentially relevant states, relational or emotional themes or experiences, noting these on a shared record visible to the client and summarising ideas and hypotheses to generate feedback. This can also be done in relation to the ‘average day’ although the concreteness of a real date might be more accessible and useful.

This process can help identify experiences that can then be explored in further detail through the mapping process described below. Attention should be paid to emotional content, interactions with others, and behaviours that may represent a form of coping or response to difficulties (this may include eating behaviour) as these may provide a starting point for further discussion.

The remainder of the session should then focus on the process of formulating or ‘mapping’ the client’s experiences. This should involve an active, collaborative discussion between the therapist and client, with the therapist drawing out a visual representation of the client’s experiences as the discussion develops, taking care to use the client’s own words (Figure 1).

But avoid talking about it for fear of how others will react

want to talk to others, get their support

Feeling ignored, not cared about

but does not last because then feel out of control and shameful, disgusting

Feeling initially soothed and emotions smothered

Binge

**Figure 1: A simple map outlining hypothetical pattern of events. See other examples in Appendix II.**

A typical starting point would be to begin with an aspect of a pattern or procedure that is offered by or accessible to the client and then track backwards or forwards in time, asking about the events that precede or follow. Clients can be given the choice about the direction they would like to focus on. When tracking it is preferable to start with a single (but typical) recent experience, rather than to talk in generalities (the latter may lead to overly vague and less personally meaningful content).

In tracking a client’s experiences, it is likely that gaps will occur (e.g. emotions or aims). The therapist should work with the client to identify and try and fill these gaps. Symbols such as question marks can be used on the diagram to indicate areas or places where the client is not sure what goes there. Where clients describe a sudden shift in feeling, leading up a behaviour, it may help to draw out this shift (see Figure 2) as a means of exploring intervening states.

Feeling Calm

?

Feeling worthless

**Figure 2: Mapping sudden shifts in state**

It is possible that clients may struggle with the labelling and naming of emotional states or feelings. Young people might especially be limited in their emotional literacy or delineation. Suggestions can be provided by the therapist in a curious and open manner (“I wonder if the feeling is a bit like … or more like …”). Where possible it is good to use the client’s own language and wording in drawing out the visual map. Where a feeling is not easily labelled, it might help instead to ask about where it is felt in the body, or even see if the client is able to draw a representation of the feeling (could draw it onto an outline of a person). Clients might also use metaphors or phrases that convey emotional content without explicitly naming emotions – these can be utilised on the map in the context of a shared understanding of their meaning.

Where clients do not explicitly refer to others or systems (e.g. school) in their lives it might be helpful to explicitly inquire about what others are doing or not doing at a particular point.

*T: So you mentioned here, being sat in your room and feeling really rubbish, I am wondering what were the rest of your family doing at this time? Is there anything you would have liked them to do at this point?*

Where clients struggle to identify states preceding or following their actions, another approach may be to ask about what the place or state or feeling they are trying to get away from or resolve when the action occurs, and likewise, what the state they are trying to get to is like. The process of mapping should focus on typical experiences in the client’s life, while also exploring exceptions and overlaps. This can be done when a particular incident, feeling or pattern has been identified, whether this is something that happens a lot, or whether this has happened before.

Young people can especially struggle with questioning. Where this is either observed or explicitly stated, the therapist can offer guesses or hypotheses and request feedback/ elaboration/ clarification from the young person. This process – of offering guesses rather than questioning but needing the young person’s input to ensure accuracy - should be made explicit, clearly framing them as guesses and offering opportunity to correct or reject.

Appendix II provides a series of example diagrams that capture particular, general patterns (adapted from Sheard et al., 2000 & Newell, 2012). These are intended as a guide for therapists and should typically not be used in therapy in the first instance, but may be helpful in some situations. For example, these diagrams can be considered where a client describes experiences that appear to match one of these diagrams. This may be helpful where a client is struggling to elaborate on their experiences. However, caution should be taken to try to avoid the situation where a client agrees a diagram fits their experience out of acquiescence. This might be avoided by being clear it is unlikely the standard diagram will fully match the client’s experiences, and using it as an opportunity to then explore what might be different for the client.

The pacing of the mapping process should be largely led by the client. Based on CAT theory, different clients (in different contexts) will have different Zones of Proximal Development (ZPD; the area between what they might achieve alone, and what they are able to do, accommodate or tolerate with the therapist’s help). As such some clients will be less able to develop and elaborate an understanding of their experiences than others. The goal of the therapist is to work within their ZPD, rather than to bring all clients to the same point (e.g. a fully completed and worked out map). It is also important to remain mindful of the client’s window of emotional tolerance in order to maintain reflective capacity. It might be useful to have a discussion at the start of therapy as to how client and therapist will be aware of when the tolerance threshold is being reached and how they can check-in on that during the course of therapy and manage it. An example might be checking-in on a 0-10 scale as to how overwhelmed the client feels and where this increases above 6, the therapist and client will dial-down the emotional focus of the conversation.

*T: I realise some of the stuff we might be talking about could be difficult. It might be upsetting, or confusing or feeling a little overwhelming. Sometimes, that is okay, and it might be something you feel you can manage, but at other times it might just feel like too much. Do you know what I mean?*

*C: Yes, sometimes I can find It difficult talking about stuff*

*T: I wonder if we could agree on a way for you to tell me if it starts to feel like too much?*

The therapist should maintain an awareness of how information related to this process might inform the reformulation or understanding of enactments, while also considering the ZPD and working to engage the young person within their current ZPD.

Some different ways clients might respond to the intervention are outlined below:

- Clients wishes to move too fast, sharing their experiences and insights but with little elaboration or connection with these experiences. For these individuals the job of the therapist is to slow the pace of the work and focus on deepening the shared understanding of the feelings and experiences. The above stance may also apply to clients who appear very avoidant of emotional content.
- A client may provide very little detail or elaboration. In this case the job of the therapist, where this is possible within the context of the therapeutic relationship, is to try and add further detail and depth to the experiences being shared. Gentle questioning, the use of cautiously presented explanatory hypotheses (“ I wonder, and I might be wrong about this, but if it’s a little bit like…”), and creative approaches such as drawing feelings or states, may help here.
- Client expresses overwhelming, difficult feelings that flood the session. Therapist would try to adopt a more cognitive stance, identifying and labelling relevant emotions/feelings without exploring these and focus on how this link together within the map/diagram.
- Client wants to push on to solutions to their problems before an understanding has been developed. Therapist may respond by slowing the pace, re-iterating the focus on understanding their experiences and difficulties, and the value of this. In some cases a client’s need for quick solutions may even form part of the map (e.g. look for quick solutions but ultimately feel disappointed when these do not emerge or do not help) but this would need to be done carefully to avoid client feeling judged.

**Identification of Reciprocal Role Procedures**

During the process of mapping the therapist can begin to work with the client to identify particular Reciprocal Roles (RRs) that are prevalent in their life. RRs are discussed in detail elsewhere (e.g. Ryle & Kerr, 2002). Briefly, they represent internalised patterns of relating, that have emerged as a result of earlier experiences, and guide the way the individuals relate to themselves and others. RRs are bipolar (e.g. see Figure 3) and tend to capture three forms of relating: self-to-self; self-to-other; other-to-self. Thus, an individual may feel rejected or shamed in response to a rejecting other (other-self), but they may also become rejecting and shaming to themselves, for example as part of negative inner dialogue (self-self).

Understanding

Validated

Criticising/ domineering

Belittled

Distant

Ignored

**Figure 3: Example Reciprocal Roles**

One method to help identify RRs is to focus on the following questions:

- How did you feel towards yourself at this time?
- How did you feel towards others at this time?
- How did you feel others were being with/responding to/ acting towards/ seeing/ relating to you at this time?
- How did you feel you were being treated by that system (e.g. school) at that time
- Is this similar or different to [other experience or pattern] – in what way?

It may also help to begin by identifying how the client felt in a given situation, before moving on to ask about what the other person was doing or not doing (or what they were doing to themselves) that led to feeling this way. By doing this the two poles of the RRs can be elucidated. When identifying RRs it is important that the pole labels are meaningful to clients and ideally deepen their awareness of the feelings present during that time. It is tempting for therapists to assume what the opposite pole will be (rejecting to rejected, abusing to abused) but these poles do not necessarily co-occur and client’s experiences may differ (rejecting to ignored/uninterested or crushed). Hence RRs should match client’s experiences as closely as possible. Therapists should provide some brief, accessible psychoeducation around RRs when they arise in the formulation, e.g. we learn how people relate to us and vice versa when we are young and then we tend to relate to ourselves and others in similar ways, often creating difficult feelings that we try and cope with but we can end up getting stuck.

**Problem Procedures**

Within CAT a number of commonly occurring, problematic procedures have been noted. Whilst these procedures do not describe every pattern a client might struggle with, they apply to some clients. Where present it may be helpful for the therapist to comment on these emerging patterns.

- **Traps**: Where negative expectations lead to behaviour which ends up confirming these expectations (I know she won’t care so I avoid her and end up feeling like she does not care)
- **Snags**: Where a particular aim is abandoned because of expected negative consequences (I do not ask for help because I know they will react negatively)
- **Dilemmas**: Where a client’s feelings are caught between two alternatives (either I am a push-over and do what others tell me, or I kick back and get angry), black or white.

It is important to identify these patterns not by their conceptual labels, but in terms of the client’s own experiences, incorporating psychoeducation relating to that individual cycle (e.g. ‘have you noticed that the way you tend to cope with feeling anxious actually leads to more anxiety?’).

**Identifying Patterns in the Room**

Whilst CAT often focuses on identifying problematic patterns and RRs within the therapy relationship, this may not be possible within the short duration of this intervention, and is not expected. Nonetheless, there may be times where it is helpful to make links between the client’s experiences and their relationship with yourself.

- Where patterns are apparent that seem likely to affect a client’s likelihood of attending the next session (e.g. a pattern of feelings other cannot help and cutting off contact from them).
- Where client’s way of relating is creating a barrier to progressing with the intervention (e.g. unwilling to engage in the intervention for fear that it might not help) it may help to reflect on how this process seems very difficult for them and ask about whether this feels like a barrier in other contexts.
- Where clients reflect positively on the experience of the intervention it may helpful to explore how their interaction with yourself differs to others they have captured in the mapping.
- Where an example from the therapy room might be more within the client’s ZPD than one outside (e.g. ‘sometimes I guess you might think I don’t really get what you’re saying- like I’m not listening properly, like it feels with school’, rather than ‘do you sometimes feel like your mum doesn’t listen either’- which the client might not be ready to explore)
- A common pattern might be around clients attempting to please or placate the therapist, saying what they believe the therapist wants to hear or what will please them. This can be a tricky pattern to label and discuss, but spotting this can be important as it may otherwise become a barrier to real change or understanding.

Perfect family

Completely belongs

Thin = athletic

I spend more time with mum, feel connected

Doesn’t last

Feel others are in control of me

I want to feel in control

People worry and I’m made to seek support from services

Feel angry, trapped and try to regain control

Controlling

I

I

I

Controlled

Pressure

Restrict my eating

Don’t attend therapy

Criticise others / therapist

Everything’s out of my control, only thing I can control is my weight

To critical reciprocal role

**Figure 4: A more detailed map outlining hypothetical pattern of events leading to barriers in the therapy room.**

**Ending Session One**

Endings are an important focus in CAT. Whilst this intervention is brief, it may be helpful to reiterate towards the end of the initial session that three more (plus one extra with their clinician/ family member) remain and reflect on feelings relating to this. It might be helpful to discuss what the client would like to get from the following sessions, or how they would like to approach them, based on session 1 and their hopes/ expectations. The brevity of this intervention may be challenging or difficult, which can be acknowledged (see below “Negative reactions to short intervention”). For some clients, where endings or related experiences (e.g. perceived rejection) have emerged as relevant feelings, it may be useful to link the ending of the session to this observation. In these instances it may help to explore how the client typically responds to endings or how endings have been handled in previous service interactions and also how this (the next intervention session) could be an opportunity to do something differently. This may include thinking aloud about why it might be difficult to attend the next session.

Following sessions one, two and three, a relevant between-session activity should be set for the young person to do in the week before the next session. The activity should link to the content of that particular session, but they would tend to involve either a) reflecting on mapping and reformulation; b) focus on monitoring for patterns or difficulties in everyday life; c) considering gaps in the map that you might want to explore next time. Planned activities should be discussed and agreed collaboratively within the session, and space should always be allowed to review how this went at the subsequent session. As with all other aspects of the intervention, the between-session activity should work within and stretch slightly the client’s ZPD. For example, if the young person is able to reflect on patterns then an activity could be to spot and note them in action, whereas if a young person finds this too much then noting experiences might be less taxing while still facilitating more focused discussion in the subsequent session.

Clients should be encouraged to reflect on the initial session and try to keep formulations or relational patterns in mind to support the work in the following session, for instance, what are the things that you’d like to take away from our conversation. For clients where a map has started to be developed they could be asked to reflect upon it and make notes on recognition and/or add to it. If the map has been developed further it can be used more actively to recognise relational pulls, patterns and new ideas of strategies that have occurred between the sessions. The client should take a copy of the map or encouraged to use their phone (if present) to take a photo to improve the availability of it.

The final 10 minutes should be kept aside to reflect on the conversation and content and help ease the transition from the session back to everyday life. This is particularly important for clients who experience distress during the session, allowing space for these clients to return to a less distressed state before the session is closed. This might be achieved through validation and normalisation that this psychological work can be difficult, and non-problem talk on non-arousing subjects or an activity (e.g. a brief card game).

**Session Two-Four**

Sessions two-four should be 60 minutes long or shorter where this has been contracted. Once again, the last 10 minutes can be set aside as time to wind-down and help the transition from the intervention to everyday life.

Session two should begin with a review and recap of the ground covered in session one, using the diagram(s) or map(s) developed in the first session as a prompt. Also any between-session tasks set in the last session should be reviewed. Where homework is not undertaken the reasons why, including whether this work was difficult or challenging, should be discussed. The diagram or map may help facilitate and exploration of the reasons behind not completing tasks. Using the map in this way may help these discussions feel non-judgemental or less emotionally charged.

The focus of the second session and beyond will then depend on the progress made in and between sessions and may involve further development of the mapping process (see below).

**Healthy Islands**

Given the length and scope of this brief intervention, it is unlikely that the focus will move to considering exits. However, it is important to engender hope during the reformulation process and consider positive and fulfilling reciprocal roles and how they can be recognised and recruited. While mapping the therapist should listen out for indications of ‘healthy islands’ – states or patterns where the young person feels a little safer or more connected or cared for. These might be present in the emerging therapeutic relationship – is the young person feeling listened to, or able to express themselves here? These can be presented on the map, maybe using a different shape or colour to highlight them. This can ensure that the young person’s strengths and opportunities are acknowledged and can provide a shared understanding of how the therapeutic contact might be helpful and areas that could be built on with others. Care should be taken not to confuse idealised or yearned for states or reciprocal roles with healthy islands – where there are ideas or experiences of ideal care, these should be explored carefully and with a compassionate understanding of their function while acknowledging their limitations, fragility or inaccessibility.

**Ending & Goodbye Summarising**

Time should be given to discussing the ending of the intervention, including any positive or negative feelings this generates. For clients with high or idealised expectations of change disappointment is likely, and time should be given to explore these feelings. Where appropriate links might be made back to the map that has been developed (e.g., “I wonder if you’re feeling a little let down even? If we look at the map I notice there has been a common pattern of feeling this way”). Clients could be encouraged to think about what they usually do with these feelings and what they could possibly do differently. Reflecting on the sessions as a whole helps to consolidate understanding and awareness and thinking about how this might continue, such as returning to the map and holding some of the conversations in mind, using writing might also help promote ongoing reflection.

In Session four, it will be helpful to allow space for the therapist to summarise and share their understanding of the young person’s difficulties, drawing together the work that has been done across the four sessions. This summary can take the form of a written ‘Goodbye letter’, as in traditional CAT, but it might also be a verbal summary. A formal goodbye letter is not required for RIDE, but some form of ‘goodbye summary’ is. The goodbye summary should encompass a) key patterns or procedures, b) key reciprocal roles or relational patterns, c) any potential exits that have been discussed or practiced, d) wider reflections (as appropriate and bearing in mind the client’s ZPD) on any challenges within the therapy, including, for example, difficult enactments of roles, with a particular focus on important steps or gains the young person has made (e.g. “I know that opening up and talking about these experiences has been incredibly hard for you; I think it says a lot about your inner strength that you have been able to overcome these barriers and start to share these experiences with me”). The summary should be offered tentatively, allowing for adjustment or correction by the young person. Following this summary, it is important to allow time for the young person to comment on what has been said and offer their own reflections.

The client should be told in session 3 that this summary or goodbye letter will be shared in the fourth session. They can also be asked to think about insights and reflections they have taken from the sessions, and potentially (if within their ZPD) note these down (e.g. as bullet points or rough notes) to share in session 4.

**Negative Reactions to Short Intervention**

From qualitative research we have seen that some individuals view their difficulties as very entrenched and can be sceptical of the idea that a short intervention will be of any use. If such concerns arise it can be noted to emphasise that such concerns are understandable, and whilst this five session intervention may not be enough to resolve or work through all of the difficult experiences they might have faced, it may nonetheless be a useful stepping stone, perhaps starting some helpful processes or changes in how they think about their experiences that could lead to bigger changes in the future. It might be worth highlighting that things have been tried before, possibly over longer time periods, and not yet been effective, and therefore the current intervention may help to ensure any future care is worth the young person’s time and effort.

For some clients the brevity of the therapy may activate or bring to the surface negative feelings about treatment (e.g. that this intervention can’t help or that nothing will help) or the possibility of change more generally (e.g. that nothing will help). Where such feelings are apparent it may be possible to comment on these and being them into the therapy room. Such feelings may be a useful indicator in thinking about patterns with others (e.g. they feel let down by others who cannot help and this feeling leads into restricting or bingeing). In these cases, links could be made between the feeling in the therapy room and these wider patterns. However, care should be taken that this does not feel blaming or judging and is done in a curious and open-minded way.

Negative feelings may also be apparent towards the end of a session, and it may be helpful to explore where these typically lead and how this situation could be different (e.g. feeling it won’t help so maybe they will miss the next session altogether, but what might it be like if they attend the next session despite this feeling).

**The fifth session**

The nature and purpose of session five should have been alluded to from the start of therapy and highlighted again and planned for during session three. The therapist and the young person will discuss the importance of handing over their joint understanding to the coordinating clinician and a family member (the latter where the young person consents) with the aim of sharing understanding and thus facilitating more effective care. The therapist will invite those appropriate and discuss with the young person in advance how this session will be managed ( e.g. who will speak about which aspects, what aspects of the map will be emphasised, will questions be fielded and by whom). There should be an emphasis on empowering the young person to share their own map or shared understanding, coupled with an awareness of their ZPD and the inherent challenges of power imbalances in this process. It may help to focus this discussion on the plan for session five around what feels most helpful to the young person and to think together, using the map as a guide, around what might be useful. It will also be important to avoid generating unrealistic expectations about what might be achieved in this fifth session, whilst still keeping an optimistic focus on what change might be possible (e.g. it is unlikely that a parent’s whole approach to parenting could be altered).

Session five should be around 60 minutes in length. Where possible it may be helpful to begin the session with the young person alone, to briefly recap on what will be discussed and how, and to check how they feel about the session before inviting the other attendees in. By involving them in this way at the start, it may be possible to help them feel like they have a role in guiding and directing the conversation based on their own needs, rather than just being a subject to be discussed by adults (a feeling they may have experienced before). Depending on the client it may also be helpful to agree on a plan around what to do if they become overly distressed or need to take time out form the session (how this might be communicated and acted on). The other attendees, who may include (but not limited to) parents, clinicians, social workers, and teachers can then be invited in, and the session proper started.

The therapist should take the role of facilitating the discussion. The session should begin by discussing the purpose of the session (which will focus primarily on how the young person is best supported and helped) and also key ground rules or boundaries for the session, especially around confidentiality. The first part of the session proper can then be spent summarising the reformulation or map that has been developed with the young person. It should be explained that the map essentially captures the feelings and relational experiences form the young person’s perspective. In this sense the discussion should not be about whether the map is right or wrong, the map captures the young person’s experience, and this is valid, even if it differs to how others experience the same situations.

The therapist should check in regularly with the other attendees to ensure they understand and to clarify any uncertainties. Depending on the stage the young person is at, it may be beneficial to allow them space to introduce and explain some aspects of the map. The discussion is focused on summarising the work and looking ahead, but done to also include wider systems and to think more specifically about their role in helping the young person. The second part of the session should focus on a review of planned exits or recommendations for future support, and a discussion of these within the group.

It is possible that the content of the map may directly relate to individuals invited to the fifth session. This issue needs to be managed with care due to the potential for some individuals to feel blamed or judged (e.g. parents who feature heavily in the map as rejecting or unsupportive). There may be times where due to the nature of a person’s impact on the client it would not be appropriate to have them present at the meeting. In other situations, making it clear the map reflects the young person’s personal experience within a range of relationships, reflecting that this may at times be difficult to hear, and keeping the focus on the young person and how they’re to be best supported, may help.

A good conclusion to the fifth session may be to produce, in addition to the existing maps, a list of recommendations, exits, or plans, for helping the young person in the future, with actions identified for the young person as well as others they interact with. This list or plan could be typed and shared, with a copy kept in the young person’s notes along with the map.

Where a young person does not want others to be involved in session five, this space can be used as an opportunity to reflect together with the young person– using the map -on what needs to change in their system to allow this kind of joint working to feel safe and useful.

**Appendix I. Alternative Psychotherapy File – Alison Jenaway**

Alternative CAT Psychotherapy File

CAT therapy is about trying to find patterns in the way you feel and behave in relationships. Once you can see the patterns more clearly, you can build on the ones which seem to work well for you, and try to change the ones which cause problems. Listed below are statements that a person might use to describe himself or herself. Please read each statement and decide how well it describes you. When you are not sure, then go with your gut instinct, rather than what you would like to be true, or how you think you should feel. If you like, you can change the words to make it even more true of you. Each set of statements goes with a type of pattern that you might recognise. The pattern is written in the box below the statements. Again, change the description of the pattern to make it more true of you if you need to, then rate the pattern in the box for how much it applies to you. There are no right or wrong answers, it is part of the process of getting to know yourself better.

Rating Scale

0 = Not at all true of me

1 = Somewhat true of me, or true of me at some times in my life

2 = Very true of me

1. I bottle up my feelings rather than speaking out -------

2. I try to please people all the time -------

3. I feel as if I do more for others than I get back in return -------

4. I find it difficult to know what I really want for myself -------

5. I am a good listener but tend not to talk about my problems to anyone -------

6. I can feel quite resentful sometimes but I don't express it -------

7. I put on an act with people, rather than just being me -------

“Too eager to please trap”

If you have said that several of these are true of you, you may be caught up in a kind of "too eager to please trap". Feeling unsure of yourself, you try to please others and do what they want, not expressing your own feelings. This can lead to you being misunderstood, or allow people to take advantage of your good nature, leading you to feel angry and even more unsure of yourself. Does this sound like you?

--------

8. If people knew the real me then they wouldn't want to be my friend -------

9. I feel as if people find me dull and boring in social situations -------

10. People don't want to include me in their groups -------

11. I worry that I am going to make a mess of everything I try -------

12. I often feel embarrassed around other people because I feel as if I am not as good as they are -------

13. I often avoid situations where I might be judged -------

14. I don't deserve good things to happen to me -------

15. I never get accused of showing off -------

“Low self esteem trap”

If you said that a lot of these statements apply to you, then you may be stuck in a "low self esteem trap". In this pattern you feel not good enough and fear judgement or criticism from others. This can lead you to avoid things, put things off or do them in a half-hearted way. This behaviour can make people irritated and they can be critical, which makes you feel even more that you are not good enough. Does this seem familiar?

--------

16. If I trust my instinct, then I'll get things wrong -------

17. I need other people to help me in a lot of areas of my life -------

18. I am not very good at taking care of myself -------

19. I tend to want other people to make the decisions about what to do and where to go -------

20. Sometimes I feel as if people treat me like a child and boss me about -------

“Depending on others trap”

If you have said that several of these statements apply to you, then you may be stuck in a “depending on others trap”. Feeling scared that you will not be able to cope, or will get things wrong, you seek out people who will do things for you and make all the decisions. At first this feels good, but it means that you do not get any practice at being independent and after a while you start to feel even less able to cope with life on your own. Does this sound like you?

---------

21. Whatever I do I have to come top, I can't accept second best

------

22. I work hard to keep everything in perfect order -------

23. I have so much to get done that there is never any time to have fun -------

24. People tell me that I push myself too hard -------

25. I am careful to control which feelings I show to other people -------

26. People know they can rely on me, if I say that I will do something then I always get it done -------

“Need to be perfect trap”

If you have said that several of these are true of you, then you may be stuck in a “need to be perfect trap”. Feeling worried about being criticized, you work hard to be perfect in all areas of your life, pushing yourself to your limit. This is exhausting, and stressful, and at some stage you might get ill or be unable to keep going. This means that you feel a failure and fear criticism even more. Does this sound like you?

-------

27. I often feel that something bad is going to happen -------

28. I am very careful with money so I do not get into debt -------

29. I tend to avoid anything that makes me anxious -------

30. I prefer to do things the same way all the time, rather than risk doing something new or different -------

31. I am really good at attending to details and checking things are correct -------

“Anxiously avoiding trap”

If you have said that several of these statements are true of you, then you may be stuck in an “anxiously avoiding trap”. Feeling scared that things might go wrong, and not sure how to cope with anxiety, you put things off or avoid them, turning down chances to do interesting things, this gradually limits your life and you never have the experience of doing something scary and managing it. Other people seem to be getting on with their lives but you are stuck in a rut, even more scared that you cannot cope.

-------

32. In relationships that matter to me, I worry about being left -------

33. I often feel that people are out to hurt me and use me -------

34. I sometimes feel that the best way to avoid being hurt is to attack first -------

35. If someone is nice to me then I wonder what they are after -------

36. It takes me a long time to trust people -------

37. I am careful and never rush into a relationship -------

“Scared of rejection trap”

If you have said that several of these statements are true of you, then you may be stuck in a “scared of rejection trap”. You have perhaps been hurt or let down by important people in your life, which makes you scared of getting attached to anyone new. You test people out and push them away to see if they really care, this can upset and annoy people and can lead to the rejection that you fear. Do you do this sometimes?

-------

38. I worry that I could really hurt someone when I get angry------

39. I have a lot of trouble accepting it when people won’t do what I want -------

40. I don't seem to have any willpower to everyday tasks -------

41. I tend to use drinking, smoking, over eating or drugs to cope with difficult feelings -------

42. How I feel about myself, and others, can switch suddenly from one state of mind to a completely different one -------

43. Sometimes the only way to cope is to blank out and switch off feelings -------

44. People find me exciting and interesting because I am so changeable -------

“Difficulty managing emotions pattern”

If you have said that several of these statements are true of you, then you may have difficulty managing your emotions. Perhaps you have grown up in a family where feelings were not allowed or seemed dangerous, you try to block feelings out but they come back even stronger and you still have not learnt how to manage them. Does this sound familiar?

These are only some of the possible patterns that people get stuck in. As you have been filling this in, you may have started to recognize a specific problem pattern of your own which none of these captures. If so, have a go at describing it in the box below:

My particular pattern:

Snags

Snags are things that stop us making changes, even though we are unhappy with the way things are. Sometimes we are limited by something inside ourselves, or the fear of who we might become if we changed. Sometimes we are limited by how others might react if we were to try and do things differently. Have a look through the following statements and tick any of them that apply to you:

1. I don’t believe that I can ever change so there is no point even trying.
2. I don’t deserve good things to happen to me.
3. Being better would be boring, I prefer to be interesting, even if it is painful sometimes.
4. I have no idea who I would be if I got better, so it is scary to change.
5. Feeling better for a bit, then getting worse again, would be even more painful than staying the way I am.
6. My family or partner would not be able to cope if I was different.
7. It would mean leaving people behind if I got better.

Is there anything else that might be keeping you stuck in difficult patterns?

Alison Jenaway

Consultant psychiatrist in psychotherapy

If you have any comments about this form, for example how easy or difficult it was to fill it in, please ask your therapist to email me at Alison.jenaway@cpft.nhs.uk

**Appendix II. Further Example CAT maps for reference**

So I try to make people love /like me by trying to be perfect and do whatever I think they want

I feel loved/liked and safe

But the feeling does not last. I get fed up of them and trying to be perfect

I feel very disappointed and let down or like a failure

It’s never enough, and I get used by others

I believe people won’t love/like me for who I am

I feel rejected, despairing unworthy of love and/or angry and cheated by others

Unmanageable feelings:

I feel bad, rejected, alone, desperate for care ad love

I reject them, or make them reject me

**‘Wanting others to like or love me’ Map**

**‘Cutting off from feelings’ Map**

Unmanageable feelings:

I feel bad, rejected, alone, desperate for care ad love

I try to cope by cutting off form these feelings, blocking them out, or making myself numb

I feel more in control

But it does not last. People get fed up with me eventually, or I feel empty and alone.

I become like a zombie

My feeling s come back and overwhelm me

**‘Striving for perfection’ Map**

Perfectly Caring

Perfectly cared for

demands on myself are unrealistic and ultimately I cannot achieve these

Get compliments about being thinner

I try control my weight, be sick

I put lots of pressure on myself

I want to make myself acceptable / loved

I have to look a certain way to fit in, but I’m ‘fat’

I’m not good enough, others are better than me

Demanding

Rejecting

Striving

Failing

Rejected

I want to be accepted / loved /admired

Perfect sister

I am bad

I should not be getting angry like this

Others feel hurt and reject me, feel I have no friends

Eventually this spills out and I get angry and criticise others

I try to keep my emotions to myself, try to control / push them down

But feel others view me as too sensitive

I want to be accepted / good enough / normal

‘FREAK’

Attacking

Attacked

Weak

Alone
