# Supplementary material for: Brief Cognitive Analytic Therapy (CAT)‐Informed Reformulation for Young People With Eating Disorders: A Case Series
Source: Clin Psychol Psychother. 2025 Apr 2;32(2):e70043. doi: 10.1002/cpp.70043 (PMC11963221; doi:10.1002/cpp.70043)
Supplement: Supplementary file 2 — Data S2 Supporting information. [file CPP-32-e70043-s002.docx]

**Supplementary File II**

**Symptom-level item scores recorded on the EDE-Q**

|  | **Participant 1** | | **Participant 2** | | **Participant 3** | | **Participant 4** | | **Participant 5** | | **Participant 6** | | **Participant 7** | | **Participant 8** | |
| --- | --- | --- | --- | --- | --- | --- | --- | --- | --- | --- | --- | --- | --- | --- | --- | --- |
|  | **Baseline** | **Post** | **Baseline** | **Post** | **Baseline** | **Post** | **Baseline** | **Post** | **Baseline** | **Post** | **Baseline** | **Post** | **Baseline** | **Post** | **Baseline** | **Post** |
| **Item 13** | 17 | 19 | 0 | 0 | 0 | 0 | 2 | 1 | 0 | 0 | 0 | 0 | 70 | 56 | 0 | 0 |
| **Item 14** | 17 | 15 | 0 | 0 | 0 | 0 | 2 | 0 | 0 | 0 | 0 | 0 | 16 | 10 | 0 | 0 |
| **Item 15** | 17 | 15 | 4 | 0 | 0 | 0 | 2 | 1 | 0 | 0 | 0 | 0 | 16 | 10 | 0 | 0 |
| **Item 16** | 2 | 4 | 5 | 3 | 0 | 0 | 0 | 0 | 26 | 27? | 1 | 0 | 0 | 0 | 20 | 20 |
| **Item 17** | 0 | 0 | 0 | 0 | 12 | 10 | 0 | 0 | 0 | 0 | 0 | 0 | 0 | 0 | 0 | 0 |
| **Item 18** | 21 | 4 | 3 | 2 | 5 | 15 | 2 | 3 | 0 | 0 | 5 | 14 | 0 | 0 | 20 | 20 |
| **Weight (kg)** | 67 | 67 | 54 | 54 | 47 | 47 | 58 | 57-59 | 43-44 | 43 | - | - | 171 | 177 | 74 | - |
| **Height (cm)** | 173 | 173 | 173 | 173 | 165 | 165 | 180 | 180 | 157 | 157 | 160 | 160 | 180 | 180 | 178 | 178 |
| **Missed period** | Y | Y | Y | Y | Y | Y | N | N | Y | Y | Y | Y | N/A | N/A | N | N |
| **How many** | All | All | 1 | 1 | 4 | 4 |  |  | 1 | 1 | 2 | 1 | N/A | N/A | N/A | N/A |
| **Pill** | Implant | Implant | N | N | N | N | N | N | N | N | N | N | N/A | N/A | Y | Y |

******Please note: All height and weights were estimates reported by the young person themselves and were not checked within the appointment by the research team.*

****** *Item questions:*

*Item 13:* *Over the past 28 days, how many times have you eaten what other people would regards as an unusually large amount of food (given the circumstances)?*

*Item 14: On how many of these times did you have a sense of having lost control over your eating (at the time you were eating)?*

*Item 15: Over the past 28 days, on how many DAYS have such episodes of overeating occurred (i.e. you have eaten an unusually large amount of food and have had a sense of loss of control at the time)?*

*Item 16: Over the past 28 days, how many times have you made yourself sick (vomit) as a means of controlling your shape or weight?*

*Item 17: Over the past 28 days, how many times have you taken laxatives as a means of controlling your shape or weight?*

*Item 18: Over the past 28 days, how many times have you exercised in a “driven” or “compulsive” way as a means of controlling your weight, shape or amount of fat, or to burn off calories?*
